# HiViT-IDS: An Efficient Network Intrusion Detection Method Based on Vision Transformer

**DOI:** 10.3390/s25061752

**Published:** 2025-03-12

**Authors:** Hai Zhou, Haojie Zou, Wei Li, Di Li, Yinchun Kuang

**Affiliations:** College of Information and Intelligence, Hunan Agricultural University, Changsha 410128, China; 2363073422@stu.hunau.edu.cn (H.Z.); zouhaojie@stu.hunau.edu.cn (H.Z.); lidi@hunau.edu.cn (D.L.); kyc@hunau.net (Y.K.)

**Keywords:** network intrusion detection, vision transformer, deep learning

## Abstract

As Internet of Things (IoT) technology sees extensive adoption in smart agriculture, smart healthcare, and smart cities, emerging systems are increasingly confronted with complex and dynamic security threats. Intrusion Detection Systems (IDS), a key technology in network security, effectively enhance IoT system safety by detecting and monitoring anomalous activities. Nevertheless, IDS relying on traditional Machine Learning (ML) technologies demonstrate limited efficacy in classifying malicious traffic. In recent years, approaches that convert network security data into image sets and leverage Deep Transfer Learning (DTL) for classification have gained rapid popularity. While these methods substantially improve detection accuracy, they also lead to increased time and resource consumption during training. To balance high detection accuracy with reduced time consumption, this study introduces an efficient intrusion detection approach based on the Vision Transformer (ViT), utilizing its powerful feature extraction capabilities to enhance performance. The proposed High-performance ViT Intrusion Detection System (HiViT-IDS) begins by transforming one-dimensional network traffic data into RGB images and leverages the ViT model’s exceptional representational power for efficient classification. Experimental results on the ToN-IoT and Edge-IIoTset datasets reveal classification accuracies of 99.70% and 100%, respectively. In comparison to existing mainstream DTL approaches, the proposed model achieves considerable reductions in training time while sustaining high performance. The findings suggest that the HiViT-IDS offers superior potential and a competitive edge in adapting to complex and dynamic network environments.

## 1. Introduction

The rapid proliferation of Internet of Things (IoT) technology in scenarios such as smart homes, precision agriculture, and smart cities has brought significant convenience to human life. However, the abundance of heterogeneous sensor devices within these systems, due to their openness and vulnerabilities, has become potential targets for attackers, raising serious concerns about IoT system security [1]. In 2023, a ransomware attack exploiting IoT device vulnerabilities targeted a subsidiary of the Industrial and Commercial Bank of China in the USA, resulting in system paralysis and severely disrupting financial operations [2]. Similarly, a cyberattack on Florida’s water supply system (USA) increased harmful elements in the water, nearly jeopardizing the health of city residents and underscoring the security risks of IoT devices in critical infrastructure [3]. Unauthorized access threatens the confidentiality of IoT devices and data, while Distributed Denial of Service (DDoS) attacks and man-in-the-middle (MITM) attacks remain among the most severe threats to IoT systems [4]. To enhance the overall security of IoT systems, traditional passive defense measures, such as firewalls and antivirus software, are increasingly inadequate against the complexity of modern network threats [5]. As an active technique for monitoring network traffic, Intrusion Detection Systems (IDS) offer comprehensive and effective decision-making support for system operators, thereby improving overall security [6]. IDS bolsters IoT network security by detecting potential threats and vulnerabilities through the monitoring and analysis of anomalous network traffic behaviors [7].

At present, IDS leveraging traditional Machine Learning (ML) and Deep Learning (DL) approaches have been extensively adopted in IoT applications [8]. Such methods work by extracting critical features from malicious attack traffic, training models on these features, and enabling the trained models to autonomously discern attack patterns, thereby accurately identifying and detecting analogous malicious traffic [9]. For example, within power industrial control systems, IDS utilizes the Random Forest (RF) approach to develop efficient classification models capable of accurately detecting malicious traffic [10]. In the context of agricultural IoT, detection techniques for DDoS that integrate Bidirectional Gated Recurrent Units (BiGRU) with Convolutional Neural Networks (CNN) demonstrate substantial enhancements in detection accuracy and robustness [11]. Moreover, for IoT botnet detection, hybrid models that integrate CNN [12] with Long Short-Term Memory networks (LSTM) reveal exceptional proficiency in a time-series analysis [13]. Recently, the transformation of one-dimensional network traffic data into image representations has gained increasing traction. Such techniques visually encode the spatiotemporal patterns of network traffic into images, thereby augmenting the model’s capacity to identify complex attack characteristics. Toldinas et al. [14] proposed the DNN+ResNet50 framework, which converts network traffic into four-channel images and applies Deep Transfer Learning (DTL), achieving accuracies of 99.80% on the UNSW-NB15 dataset [15] and 99.70% on the BOUN DDoS dataset [16]. Nevertheless, its training time on UNSW-NB15 extends to 64,839.1 s, underscoring the need for enhanced training efficiency. The ELETL-IDS [17] framework, employing DTL, integrates five pre-trained CNN such as VGG16, Inception, and EfficientNet to attain 100% accuracy on the CIC-IDS-2017 dataset [18] and CSE-CICIDS-2018 dataset [19]. However, this method incurs considerable training time costs, requiring up to 36,366 s on CIC-IDS-2017 [18], thus demonstrating its constraints in resource-limited environments. Similarly, the TL-CNN-IDS approach proposed by Yan et al. [20] utilizes transfer learning to convert non-image network intrusion datasets into image datasets, integrating VGG16, Inception, and Xception as classifiers. This method achieves promising performance on the CIC-IDS-2017 [18] and NSL-KDD [21] datasets. Although DTL-based intrusion detection approaches achieve high performance, their extensive training time hinders real-time response to emerging attacks in dynamic threat environments.

While IDS based on traditional ML and DL techniques are widely deployed, they often face performance challenges. IDS solutions utilizing DTL achieve high accuracy but are frequently constrained by prolonged training times. Addressing the critical need to balance high performance with reduced training time, this study proposes a High-performance ViT Intrusion Detection System (HiViT-IDS). Unlike DTL techniques, the HiViT-IDS not only achieves superior accuracy, but also significantly reduces training time. The primary contributions of this work are as follows:

- During Data Pre-processing, unnecessary features are eliminated, and one-dimensional data are converted into images, which are then input into a ViT-based model for the efficient detection of malicious traffic.
- The proposed model is compared with mainstream DTL-based IDS approaches. It demonstrates competitive accuracy while substantially reducing training time, providing a significant advantage in complex network environments.
- On the ToN-IoT and Edge-IIoTset IoT security datasets, the HiViT-IDS achieves detection accuracies exceeding 99%.

The structure of this paper is organized as follows: Section 2 provides a review of related work. Section 3 introduces the proposed IDS architecture. Section 4 presents a detailed discussion and analysis of the model’s performance. Finally, Section 5 summarizes the full paper, discusses the limitations of the model, and looks at future research directions.

## 2. Related Work

In recent years, IDS based on ML and DL technologies have demonstrated potential in addressing complex network threats, garnering significant attention from researchers and experts. CNN-AttBiLSTM [22] is a DDoS attack detection approach that combines attention mechanisms with CNN-Bidirectional Long Short-Term Memory (BiLSTM). RF and Pearson algorithms are used to filter key features, while CNN and BiLSTM extract spatiotemporal features, further refined through an attention mechanism. On the CIC-DDoS2019 dataset [23], the method achieved a detection accuracy of 95.67%. While its effectiveness has been demonstrated, its performance leaves scope for enhancement. HDL [24] is a hybrid DL approach aimed at bolstering the security of agricultural IoT systems. This model integrates BiGRU and CNN, optimizing hyperparameters with an enhanced Black Widow algorithm for superior performance. Results from experiments on the ToN-IoT [25] and Edge-IIoTset [26] datasets indicate that HDL enhances detection accuracy and efficiency, making it well-suited to intricate agricultural IoT settings. Qureshi, S. et al. proposed a lightweight scheme, GuardDroid, for efficiently identifying multiple classes of malware in IoT infrastructure [27]. PSO-DNN [28] is tailored to meet the security requirements of the Internet of Medical Things (IoMT), utilizing the particle swarm optimization (PSO) algorithm to refine the DNN model hyperparameters. Experimental results on the IoMT dataset [29] show that PSO-DNN attained a detection accuracy of 96%, significantly improving IoMT security. IDS leveraging traditional ML/DL technologies have demonstrated effectiveness in certain areas; however, their accuracy falls short of expectations, limiting their capability to handle the dynamic and complex network threat environment.

DTL techniques have gained widespread application in intrusion detection, showcasing outstanding detection accuracy. Li et al. [30] introduced a network intrusion detection method based on DTL, which converts network traffic data into RGB images for input into five CNN models, such as VGG16 and VGG19. By incorporating confidence-weighted ensemble strategies and hyperparameter optimization algorithms, this method achieves remarkable performance on the CIC-IDS-2017 [18] and Car-Hacking datasets [31]. Nonetheless, its training time of 2490.5 s significantly hinders its adaptability in complex network scenarios. DTL-IDS [32] transforms the Edge-IIoTset dataset [26] into image data and applies genetic algorithms (GA) to optimize the hyperparameters of seven CNN models, such as VGG16, VGG19, and Inception, selecting the top five models for ensemble learning. While the method demonstrates notable performance on the Edge-IIoTset dataset [26], ELETL-IDS [17] employs an ensemble of five pre-trained CNN models, such as VGG16, Inception, and EfficientNet, attaining 100% detection accuracy on the CIC-IDS-2017 [18] and CSE-CICIDS-2018 datasets [19]. However, this method is exceptionally time-intensive, requiring 36,366 s of training on the CIC-IDS-2017 dataset [18], which challenges its applicability in rapidly shifting threat landscapes. VGG16-PSO [33] converts the NSL-KDD dataset into image data for input into a transfer learning-based VGG16 model, optimizing hyperparameters via the PSO algorithm. While effective, the time-intensive hyperparameter optimization process presents a pressing challenge amidst the rapid evolution of network attack techniques. DTL-based IDS approaches achieve high detection accuracy; however, their extensive training time remains a major challenge in dynamic and complex network environments.

While DTL techniques exhibit immense potential in network intrusion detection, their superior performance frequently entails substantial time costs. The computational requirements of integrating multiple migration learning models and hyperparameter optimization lead to high resource consumption, which is a huge challenge in IoT environments. To tackle this challenge, this study introduces the HiViT-IDS. Compared to traditional DTL methods, HiViT-IDS maintains high detection accuracy while significantly reducing resource consumption and training time, making it more adaptable to IoT network environments and offering an efficient IDS solution.

## 3. HiViT-IDS

**Figure 1:**
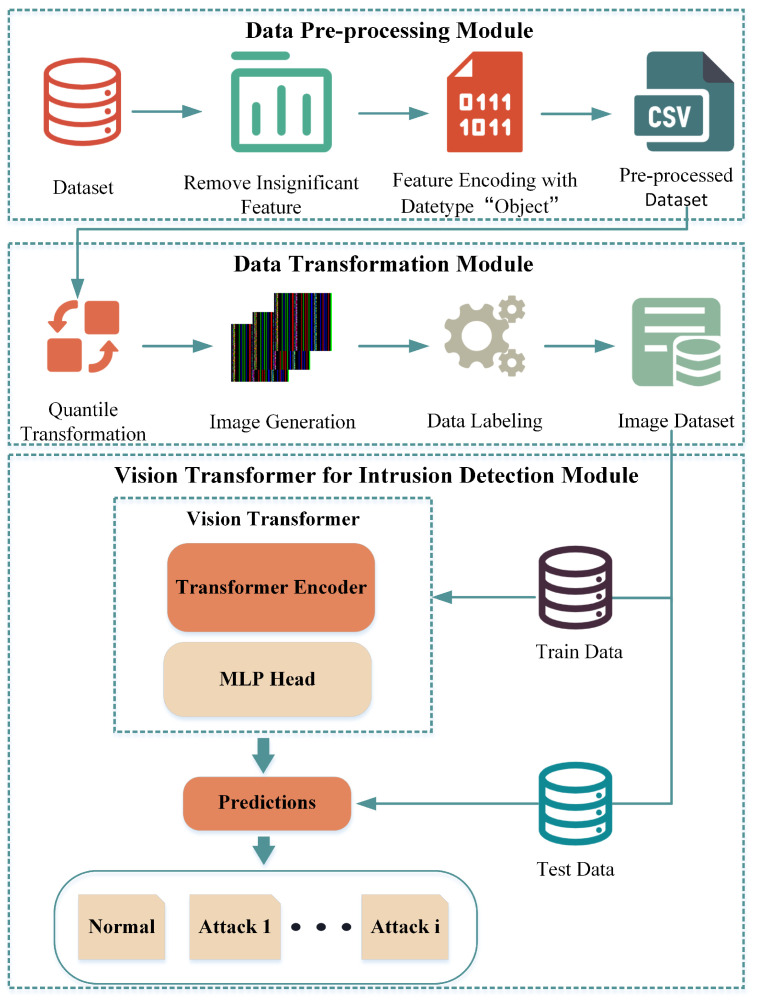
The architecture of the HiViT-IDS.

The proposed IDS is illustrated in Figure 1 and consists of three main modules: The first module involves Data Pre-processing, which includes the removal of irrelevant feature values and the encoding of the Object feature, resulting in a Pre-processed dataset. The second module is Data Transformation, wherein the Pre-processed dataset is converted into a set of images using Quantile normalization techniques [34], followed by the labeling of these images to form a complete image dataset. The third module is the training and testing of the ViT model, where 70% of the dataset is used as the training set, 10% of the data are used as the validation set to train the ViT model, and finally, the model is tested using the remaining 20% of the test set and the classification results are output.

### 3.1. Dataset Description and Data Pre-Processing Module

**Figure 2:**
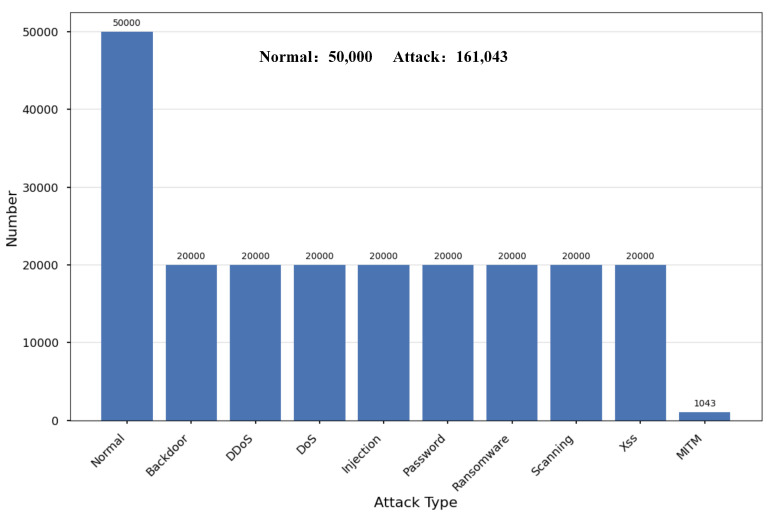
Distribution of Normal and Attack types in the ToN-IoT dataset.

In our experiments, we utilized two well-known IoT datasets: ToN-IoT and Edge-IIoTset. Both datasets are derived from IoT-enabled smart devices and encompass a wide range of network threats prevalent in IoT systems. The ToN-IoT dataset, a next-generation IoT dataset, was generated in an Industry 4.0 environment [25]. Character-based features (such as 27 unfriendly features like ‘weird_notice’ and ‘http_version’) were digitally encoded using Label Encoding in the sklearn library [35]. Following Data Pre-processing, the ToN-IoT dataset consists of 197,043 rows and 43 columns, with the distribution illustrated in Figure 2.

**Figure 3:**
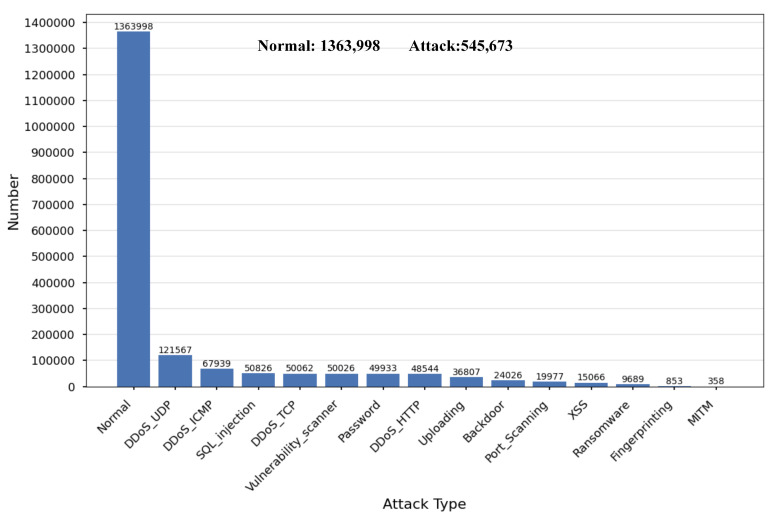
Distribution of Normal and Attack types in the Edge-IIoTset dataset.

The Edge-IIoTset dataset was generated based on a real-world IoT environment and includes 14 attack categories related to IIoT connectivity protocols [26]. During the Data Pre-processing, 815 duplicate rows were removed and 16 unnecessary features, such as ‘frame.time’, ‘ip.dst_host’, ‘ip.src_host’, and ‘arp.src.proto_ipv4’, were discarded [32]. Subsequently, the character-based features were digitally encoded using Label Encoding from the sklearn library. After Data Pre-processing, the Edge-IIoTset dataset consists of 1,909,671 rows and 96 columns, with its distribution depicted in Figure 3.

### 3.2. Data Transformation Module

Following Data Pre-processing, the dataset was further transformed into a collection of images. In the first stage of data transformation, we applied Quantile normalization techniques [34], scaling the feature values to the range of 0–255. The calculation principle is shown in Equation (1):

(1) X=(X−Min(X))/(Max(x)−Max(X))×255.

**Figure 4:**
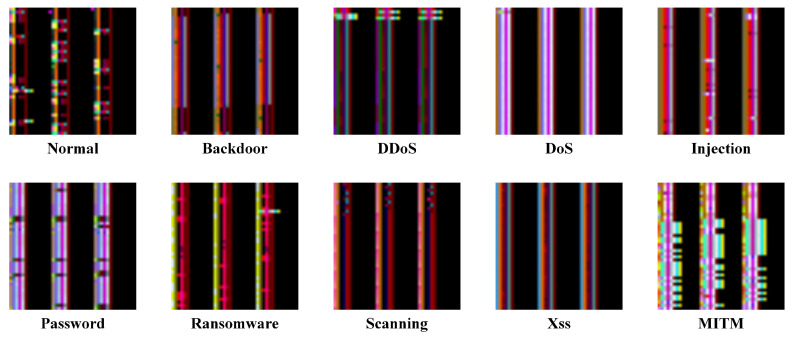
The distribution of data types in the ToN-IoT dataset converted into images.

**Figure 5:**
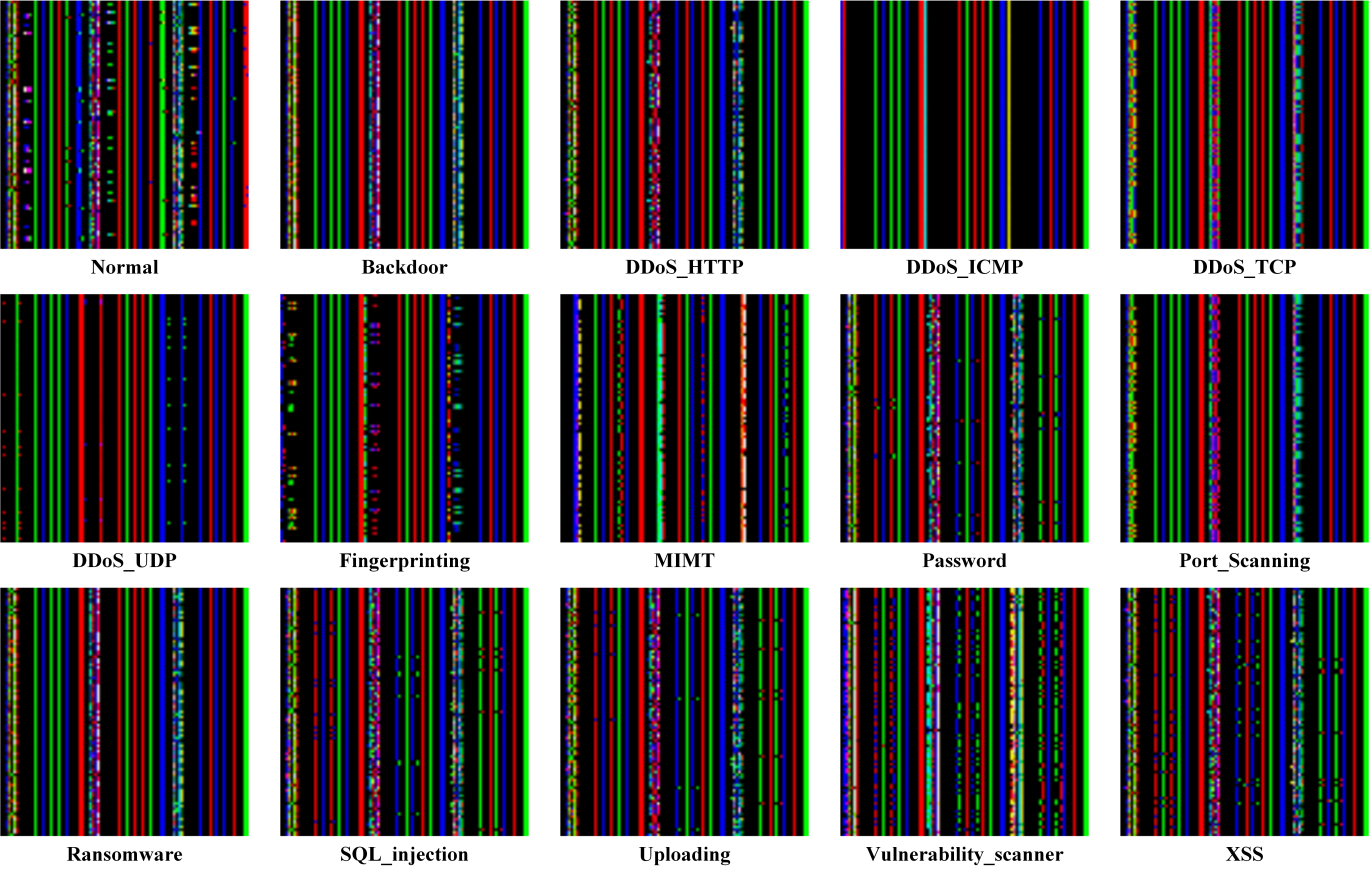
The distribution of data types in the Edge-IIoTset dataset converted into images.

Upon completion of the normalization process, the dataset was grouped by similar samples and converted into image blocks. Specifically, the preprocessed ToN-IoT dataset, which consists of 42 features, was transformed such that every 126 consecutive rows of features were converted into an image block of size 42 × 42 × 3. For the preprocessed Edge-IIoT dataset, containing 95 features, every 285 consecutive rows of features were transformed into an image block of size 95 × 95 × 3. The converted image blocks are all three-channel square color images (red, green, and blue). For example, in the case of the ToN_IoT dataset, the first 1764 (42 × 42) samples of each block are mapped to channel 1, the next 1764 samples to channel 2, and the last 1764 samples to channel 3. All samples are typically mapped to the RGB channels of the image. The data-to-image matrix conversion was implemented using the OpenCV library. Since the dataset is grouped into image blocks based on similar samples, similar image blocks are organized into the same folder to facilitate classification and labeling. For instance, in the ToN-IoT dataset, similar DoS image blocks are grouped into a folder named ‘DoS’. The data are converted into RGB image blocks, which can improve the learning ability of the model [17]. The resulting image datasets are shown in Figure 4 and Figure 5. For instance, in the ToN-IoT dataset, the images of the Normal class and the Attack class exhibit visually distinct differences: the Normal class displays a cross-hatched and star-shaped distribution, whereas the DoS attack class shows a predominantly striped distribution.

### 3.3. ViT Classifier Module

The Transformer model was first introduced by Ashish et al. [36] in 2017. Subsequently, in 2020, the Google team applied the Transformer architecture to the field of image classification. Although this was not the first work to employ Transformer models in computer vision, it became a milestone in the field of image classification due to its remarkable performance [25]. The Transformer captures long-term dependencies using a self-attention layer, while the converter asynchronously learns diverse interactions between spatial locations and input processes, enabling faster model performance. Additionally, ViT has been shown to outperform convolutional models slightly [37]. Inspired by the conversion of network traffic data into image sets, we applied ViT to network intrusion detection tasks and achieved outstanding performance on the ToN-IoT and Edge-IIoT datasets.

**Figure 6:**
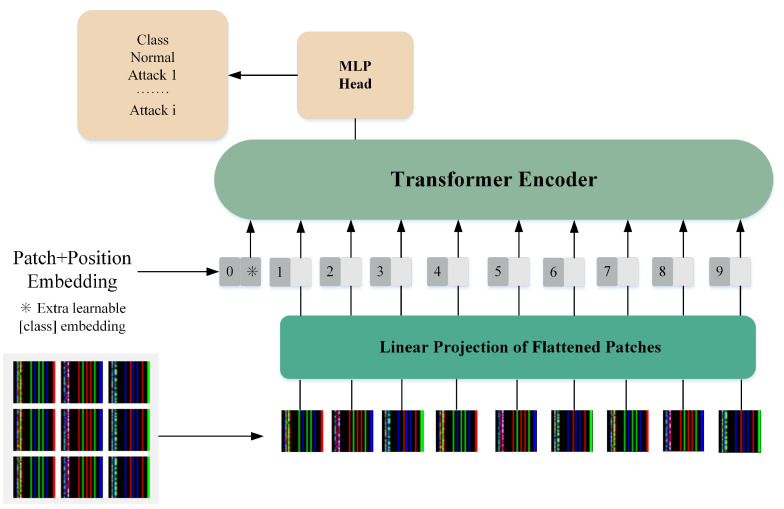
Application of the ViT model in the proposed IDS.

After the network security datasets are transformed into image sets, the images are resized to 224 × 224 × 3 and fed into the ViT model for malicious traffic detection. The core structure of ViT includes a multi-head attention mechanism and a Transformer encoder, as illustrated in Figure 6.

Initially, the input images are partitioned into multiple smaller patches of fixed size, with each patch being linearly mapped to a one-dimensional vector. These vectors, along with a classification token and positional encoding, are then input into the Transformer encoder. The Transformer encoder extracts features from the input vectors and ultimately outputs the classification results. The hyperparameter configuration of ViT used in this work is detailed in Section 4.1.

## 4. Result and Analysis

In this section, we will discuss and analyze the proposed HiViT-IDS with respect to the ToN-IoT and Edge-IIoTset datasets.

### 4.1. Experimental Environment and Model Hyperparameters

This study utilizes the TensorFlow 2.15 framework in Python 3.11.8 to build the ViT model. In the experiments, the proposed model is evaluated on a machine equipped with an intel (Santa Clara, CA, USA) 8-core E5-2686 V4 processor and an GeForce (NVIDIA, Santa Clara, CA, USA) RTI 2080TI graphics card with 23.6 GB of memory. The hyperparameter configuration for the proposed ViT model is presented in Table 1. To ensure fairness in the experiments, all comparison models are trained for a uniform number of epochs, set to 55.

### 4.2. Evaluation Metrics

We evaluate the proposed IDS model using six metrics. The four metrics Accuracy, Precision, Recall, and F1 are commonly used for performance evaluation in ML or DL classification tasks [38]. The parameters for calculating the four metrics Accuracy, Precision, Recall, and F1 are detailed as follows [39]:

- False Positive (FP): Denotes the situation in which the system mistakenly identifies normal behavior or traffic as malicious activity or an attack.
- False Negative (FN): Indicates a situation where the system fails to identify genuine attacks or malicious activities, misclassifying them as normal behavior.
- True Positive (TP): Represents the instance where the system accurately detects real attacks or malicious activities and appropriately classifies them as threats.
- True Negative (TN): Describes the case in which the system accurately recognizes normal behavior or traffic as non-malicious.

Training efficiency is a critical factor for DL models [40]. To assess the training efficiency of the model, we introduce the training time and testing time as performance metrics for the IDS. Accuracy refers to the proportion of correctly identified samples among all samples in the dataset [41], and its calculation is given by Equation (2):

(2) $$Accuracy=\frac{TP+TN}{FP+FN+TP+TN}.$$

Precision refers to the ratio of correctly classified attacks to the total number of predicted attacks [41], and its calculation is given by Equation (3):

(3) $$Precision=\frac{TP}{TP+FP}.$$

Recall refers to the ratio of correctly predicted attacks to the total number of attacks in the test set [41], and its calculation principle is given by Equation (4):

(4) $$Recall=\frac{TP}{TP+FN}.$$

F1, as a balanced metric for the model, is the harmonic mean of Recall and Precision [42], and its calculation principle is given by Equation (5):

(5) $$F1=\frac{2\times Precision\times Recall}{Precision+Recall}.$$

Train time refers to the amount of time the model spends during training (in seconds), while Test time denotes the time the model takes to make predictions on the test set (in seconds).

### 4.3. Results and Analysis on ToN IoT Dataset

**Figure 7:**
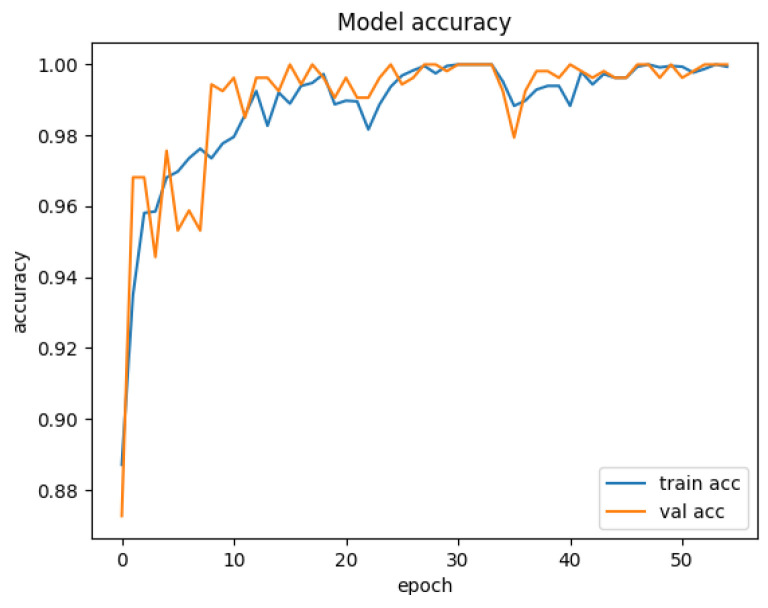
Accuracy curve of the HiViT-IDS on the ToN-IoT dataset.

**Figure 8:**
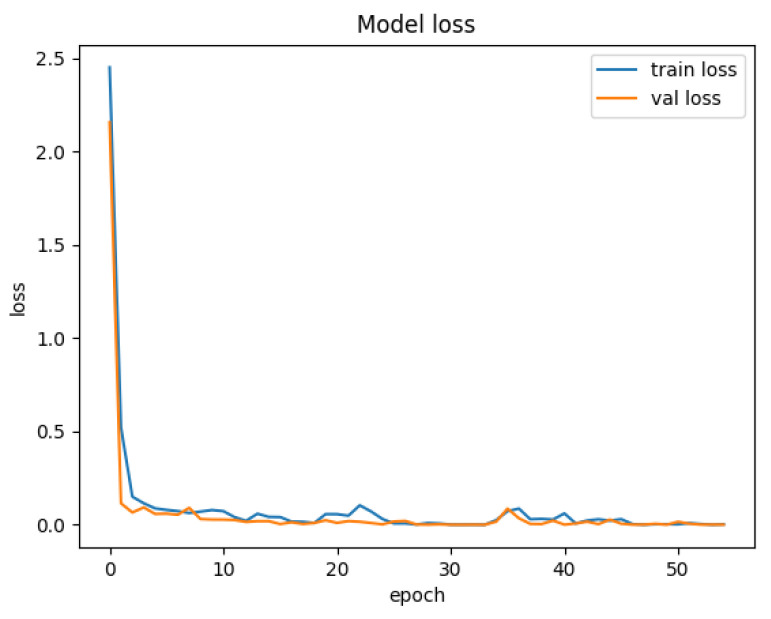
Loss curve of the HiViT-IDS on the ToN-IoT dataset.

Figure 7 depicts the training and validation accuracy curves of the HiViT-IDS on the ToN-IoT dataset. The accuracy improves rapidly during the first few training epochs and continues to approach 1 after 40 epochs. Figure 8 shows the training and validation loss curves for the model on the ToN-IoT dataset, where the loss begins to plateau after 40 epochs.

According to Table 2, the HiViT-IDS achieves the highest levels in Accuracy (99.70%), Precision (99.71%), Recall (99.70%), and F1 (99.70%), surpassing all other comparative CNN models. Additionally, the performance of Xception and CNN is relatively close, yet still inferior to our model, particularly in the consistency between Precision and Recall. Our model demonstrates a significantly improved training efficiency, with a training time of 53.49 s, much lower than that of the other models. The CNN model takes 192.79 s, approximately 3.6 times longer than our model. EfficientNetB7 exhibits the longest training time at 891.26 s, which is 16.7 times longer than ours.

As shown in Table 3, DTL-IDS [32] achieves a perfect score of 100% across all performance metrics (Accuracy, Precision, Recall, and F1), theoretically representing the optimal classification performance. However, this performance comes at the cost of a very high time expenditure. The HiViT-IDS performs excellently with Accuracy (99.70%), Precision (99.71%), Recall (99.70%), and F1 (99.70%), slightly lower than DTL-IDS [32] and ELETL-IDS [17], but demonstrating a significant advantage in terms of time and resource consumption. The performance of ELETL-IDS [17] is also high (99.89%), outperforming the TL-CNN-IDS [20] and Li [30] models, but still falls short of DTL-IDS [32]. The training time of DTL-IDS [32] is 22,442.83 s, the highest among all models, indicating that its performance improvement comes with a substantial computational cost. In contrast, the training time of our model is only 53.49 s, far lower than that of other models. TL-CNN-IDS [20] has a training time of 2475.19 s, approximately 46 times longer than ours, and ELETL-IDS [17] has a training time of 8103.83 s, about 151 times longer. The DTL-based intrusion detection systems, including DTL-IDS [32], TL-CNN-IDS [20], Li [30], and ELETL-IDS [17], utilize optimization algorithms to adjust hyperparameters. While these approaches demonstrate impressive results, their prolonged training process significantly hinders timely adaptability, increasing the potential for security risks in highly dynamic and complex network scenarios. The HiViT-IDS achieves the optimal balance between high performance and resource efficiency, maximizing both training and testing time efficiency, making it the best choice in terms of both performance and resource consumption. In addition, the CNN-LSTM [43] and Transformer [44] models are subjected to additional comparative experiments, and the realization results indicate that CNN-LSTM and Transformer are 2.01% and 4.35% lower than HiViT-IDS in terms of accuracy, respectively, while HiViT-IDS is significantly lower in training time than both CNN-LSTM and Transformer.

**Figure 9:**
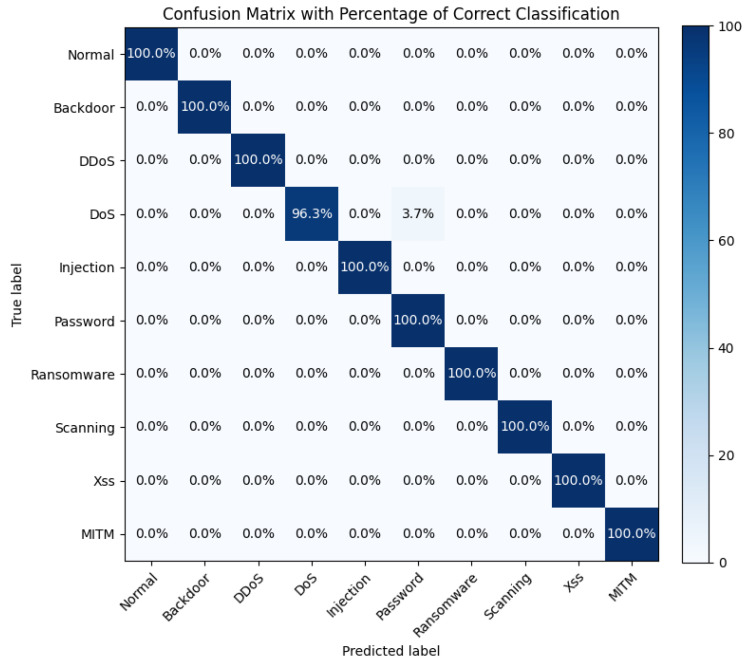
Confusion matrix of the HiViT-IDS on the ToN-IoT dataset.

Figure 9 presents the confusion matrix of the HiViT-IDS on the ToN-IoT dataset, where the performance in the DoS classification is suboptimal.

The reasons for the superior performance and time performance of HiViT-IDS over CNNs and RNN models may lie in the fact that, first, CNNs use convolutional filters to analyze images, focusing on local features [45]. In contrast, ViTs utilize a transformer architecture to process an image as a sequence of patches and apply self-attention mechanisms to understand global relationships in the image. Second, traditional RNN model training is iterative and sequential, resulting in exceptionally long training times [37]. In contrast, ViT training is parallel, allowing all functions to be trained at the same time, which significantly improves computational efficiency and reduces model training time.

### 4.4. Results and Analysis of Edge-IIoTset Dataset

**Figure 10:**
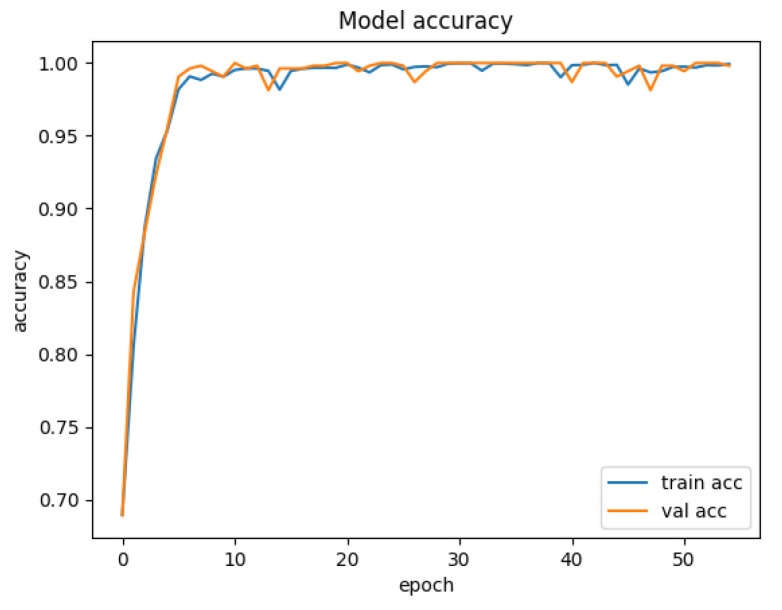
Accuracy curve of the HiViT-IDS on the Edge-IIoTset dataset.

**Figure 11:**
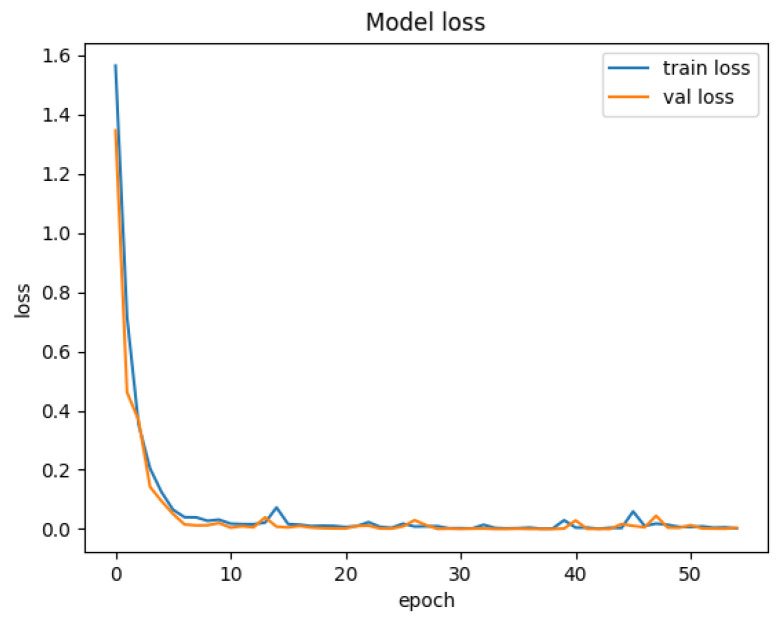
Loss curve of the HiViT-IDS on the Edge-IIoTset dataset.

To further validate the effectiveness of the HiViT-IDS, we conducted supplementary experiments on the Edge-IIoTset dataset. Figure 10 illustrates the accuracy curve of the HiViT-IDS on Edge-IIoTset, where the accuracy curve converges quickly in the early epochs and gradually stabilizes around the 10th epoch, with both training accuracy and loss approaching 1. Figure 11 presents the training loss and validation loss curves of the HiViT-IDS on Edge-IIoTset. As shown in the figure, both the training loss (train loss) and validation loss (val loss) decrease rapidly during the first few epochs and then stabilize at lower levels.

The performance comparison of the HiViT-IDS and the CNN baseline model on the Edge-IIoTset dataset is presented in Table 4. The HiViT-IDS achieves 100% in Accuracy, Precision, Recall, and F1, delivering optimal performance and demonstrating its strong classification capability. InceptionV3 and VGG19 also perform well, with Accuracy values of 99.40% and 98.95%, respectively, which are close to optimal, and their Precision and Recall scores are relatively high. Xception and VGG16 exhibit noticeably inferior performance, especially Xception, with an Accuracy of 72.04% and a Precision of only 51.90%. In terms of time and resource consumption, the HiViT-IDS has a training time of 160.91 s, making it one of the least time-consuming models, second only to VGG19 at 455.74 s and CNN at 518.94 s. EfficientNetB7 has the longest training time, reaching 3194.08 s, resulting in significant resource consumption. The model with the best performance and efficiency is the HiViT-IDS, which not only achieves the highest classification performance, but also significantly reduces both training and testing times.

In Table 5, on the Edge-IIoTset dataset, both DTL-IDS [32] and the HiViT-IDS achieve 100% in Accuracy, Precision, Recall, and F1, demonstrating identical optimal performance. ELETL-IDS [17] ranks second, with an Accuracy of 99.96%. Li [30] and TL-CNN-IDS [20] achieve Accuracy values of 99.85% and 99.80%, respectively, which are also close to optimal but slightly inferior to DTL-IDS [32] and ours. The HiViT-IDS has a training time of 160.91 s, significantly lower than that of other models, making it the most efficient in terms of training time. DTL-IDS [32] has a training time of 32,512.08 s, nearly 200 times longer than ours, resulting in extremely high resource consumption. DTL-IDS [32], TL-CNN-IDS [20], Li [30], and ELETL-IDS [17] integrate optimization algorithms to refine model hyperparameters. However, this process incurs considerable time overhead during the training phase. The results confirm that the HiViT-IDS outperforms in both performance and time efficiency, making it the optimal solution for high performance and efficiency. While DTL-IDS [32] achieves classification performance equivalent to ours, its high training and testing times limit its applicability. In addition, CNN-LSTM [43] and Transformer [44] were subjected to additional experiments, which showed that HiViT-IDS was at least 4% ahead in terms of accuracy. It also significantly reduces training time.

**Figure 12:**
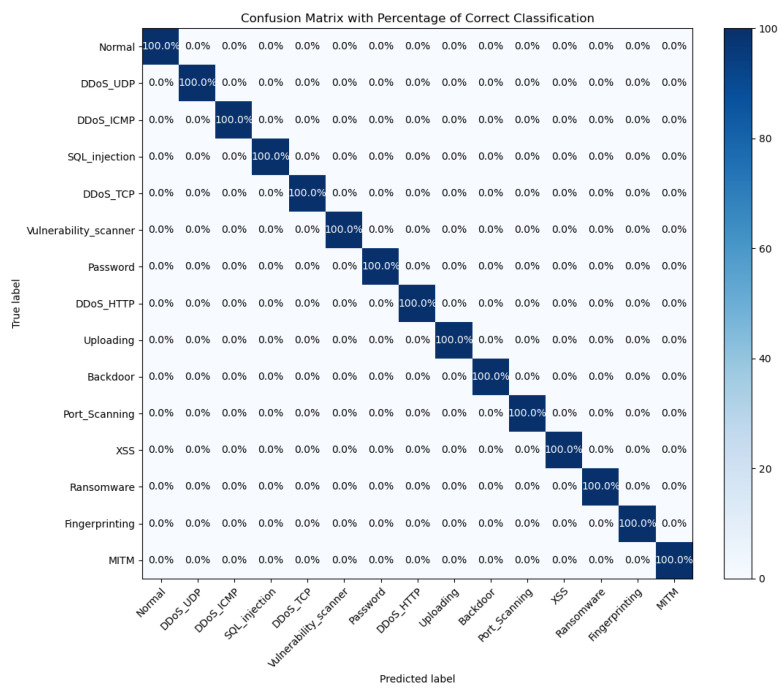
Confusion matrix of the HiViT-IDS on the Edge-IIoTset dataset.

The confusion matrix of the HiViT-IDS on the Edge-IIoTset dataset is shown in Figure 12.

The possible reasons for the proposed model taking less time for training and testing are: The network intrusion datasets are converted into image sets for input into ViT. The ViT outperforms the limitations of RNN by using self-attention for attack classification and replay attack detection [45]. The proposed HiViT-IDS demonstrates outstanding performance on the larger Edge-IIoTset dataset. This may be attributed to the fact that, on smaller datasets, the lower input information density hampers the ViT’s ability to fully extract features. In contrast, larger datasets significantly enhance the ViT’s performance by increasing the information density [46].

## 5. Conclusions

Applications such as smart farming and smart healthcare demonstrate the rapid development of IoT technologies, significantly improving the quality of human life and advancing societal progress. However, the widespread integration of interconnected devices has also created opportunities for hackers and malicious attackers, making it imperative for IoT systems to enhance their security defenses. IDS, as a security technology that monitors network traffic, can effectively identify and detect malicious activities and network attacks. In recent years, IDS solutions based on DTL have gained traction. These methods typically convert one-dimensional network traffic features into image sets, which are then processed by CNN with pre-trained weights. While these approaches exhibit excellent performance, the model training process is time-consuming and resource-intensive, which may hinder their adaptability in complex and dynamic network environments, delaying the detection of malicious attacks. To address these challenges, we propose a ViT-based network intrusion detection solution and validate it through experiments on two well-known IoT security datasets, ToN-IoT and Edge-IIoTset. Experimental results show that, compared to mainstream DTL models, our model achieves 99.7% and 100% accuracy on the ToN-IoT and Edge-IIoTset datasets, respectively. Moreover, our model significantly reduces training time and resource consumption, showcasing its competitive edge in complex network environments.

Although the HiViT-IDS model demonstrates strong performance on two IoT datasets, this study does not address adversarial sample testing for ViT. Future work will incorporate adversarial testing to enhance the model’s robustness. In addition, incremental learning strategies are a direction worth exploring in the future in the face of ever-changing cyber threats. 

## Figures and Tables

**Table 1:** Hyperparameter configuration for training the HiViT-IDS model.

Hyperparameter	Value	Hyperparameter	Value
input_shape	[224, 224, 3]	patch_size	8
learning_rate	0.001	num_patches	256
num_epochs	55	projection_dim	64
batch_size	32	num_heads	2
image_size	128	transformer_layers	1
weight_decay	0.0001	mlp_head_units	[2048, 1024]

**Table 2:** Performance comparison between the HiViT-IDS and CNN on the ToN-IoT dataset.

Model	Accuracy (%)	Precision (%)	Recall (%)	F1 (%)	Train Time (s)	Test Time (s)
CNN	99.40	99.41	99.10	99.35	192.79	1.70
VGG19	98.49	98.24	98.49	98.35	148.58	0.99
VGG16	98.49	98.06	98.49	98.21	143.17	0.87
InceptionV3	98.80	98.99	98.80	98.83	219.00	1.82
EfficientNetB7	98.80	98.85	98.80	98.80	891.26	7.28
Xception	99.10	98.54	99.10	98.81	143.39	1.50
HiViT-IDS	99.70	99.71	99.70	99.70	53.49	0.89

**Table 3:** Performance comparison of the HiViT-IDS with current mainstream DL approaches on the ToN-IoT dataset.

Model	Accuracy (%)	Precision (%)	Recall (%)	F1 (%)	Train Time (s)	Test Time (s)
DTL-IDS [32]	100.00	100.00	100.00	100.00	22,442.83	5.19
TL-CNN-IDS [20]	99.69	99.69	99.69	99.69	2475.19	2.01
Li [30]	99.79	99.79	99.79	99.79	4528.93	1.89
ELETL-IDS [17]	99.89	99.89	99.89	99.89	8103.83	2.64
CNN-LSTM [43]	97.69	95.72	96.01	95.82	629.40	3.61
Transformer [44]	95.35	93.46	94.82	94.14	2502.59	14.05
HiViT-IDS	99.70	99.71	99.70	99.70	53.49	0.89

**Table 4:** Performance comparison between the HiViT-IDS and CNN on the Edge-IIoTset dataset.

Model	Accuracy (%)	Precision (%)	Recall (%)	F1 (%)	Train Time (s)	Test Time (s)
CNN	93.85	93.87	93.85	93.84	518.94	1.3
InceptionV3	99.40	99.17	99.40	99.24	569.89	2.1
VGG16	77.81	60.85	77.81	68.20	484.73	2.7
VGG19	98.95	98.93	98.95	98.93	455.74	2.1
InceptionResNetV2	95.43	96.21	95.43	94.95	1405.88	3.4
EfficientNetB7	98.78	98.78	98.78	98.78	3194.08	4.3
Xception	72.04	51.90	72.04	60.33	506.06	3.2
HiViT-IDS	100	100	100	100	160.91	1.4

**Table 5:** Performance comparison of the HiViT-IDS with current mainstream DL approaches on the Edge-IIoTset dataset.

Model	Accuracy (%)	Precision (%)	Recall (%)	F1 (%)	Train Time (s)	Test Time (s)
DTL-IDS [32]	100	100	100	100	32512.08	6.15
TL-CNN-IDS [20]	99.80	99.81	99.80	99.81	3911.99	2.32
Li [30]	99.85	99.86	99.85	99.85	7821.13	4.15
ELETL-IDS [17]	99.96	99.97	99.96	99.97	10993.93	4.75
CNN-LSTM [43]	94.92	88.35	77.31	78.27	5833.60	83.59
Transformer [44]	95.92	88.84	88.10	88.47	9727	214.6
HiViT-IDS	100	100	100	100	160.91	1.4

## Data Availability

The data presented in this study are available in [25,26].
